# Impact of child marriage on adolescents' health and socioeconomic development: an essay on Ethiopian perspective

**DOI:** 10.11604/pamj.2025.51.84.36033

**Published:** 2025-08-01

**Authors:** Andamlak Gizaw Alamdo, Adom Manu, Agnes Millicent Kotoh

**Affiliations:** 1 Department of Health Service Management, Health Promotion, Reproductive Health and Nutrition, School of Public Health, Saint Paul's Hospital Millennium Medical College, Addis Ababa, Ethiopia,; 2 Department of Population, Family and Reproductive Health, School of Public Health, College of Health Sciences, University of Ghana, Legon, Ghana

**Keywords:** Child marriage, impact, adolescent, health, Ethiopia

## Abstract

Early marriage is a worldwide health concern that is prevalent in Southern Asia, South America, and Sub-Saharan Africa. Early marriage has been associated with numerous adverse outcomes, including an increased risk of unintended pregnancy, poor economic status, discontinuation of education, sexually transmitted infections, higher divorce rates, adverse pregnancy outcomes, maternal and child mortality, and various negative physical and social consequences for young women and their children. Child marriage in Ethiopia is one of the highest rates in the world. Although the country has shown an overall reduction in the prevalence of child marriage, the problem is still striking and demands the attention of all stakeholders who are working on children's and adolescents' health and well-being. The most challenging side of early marriage in Ethiopia is that it is linked with a diverse and highly deep-rooted issues such as religion, culture, ethnicity, and other conditions. Examining the customs surrounding child marriage, and involving the society, families, and the young people in the planning, implementation and evaluation of all programs concerning child marriage is critical.

## Essay

Early marriage is a worldwide health concern prevalent in Southern Asia, South America, and Sub-Saharan Africa. Any official marriage or informal relationship in which one or both parties are under the age of 18 is classified as child marriage, and it is an extreme example of gender inequality [1]. Every year, around 12 million girls marry before they reach the age of 18. Gendered social norms and unequal connections between men and women are the basis of the practice. Poverty, a lack of education, and social and economic uncertainty exacerbate the problem [2]. Marital age is a period of maturation that marks the end of some changes in education, employment, and social involvement, as well as the beginning of socially appropriate times for sexual intercourse and childbearing [3,4].

Early marriage has been linked to a higher risk of unintended pregnancy, poor economic status of women, discontinuation of education, risk of sexually transmitted infection, higher divorce rates, a variety of negative physical and social consequences for young women and their children, adverse pregnancy outcomes, as well as an increased risk of death for the mother and her child [5,6]. Girls who marry at a young age lose their progress in education, have a low potential for paid employment in the work industry, and are thus largely employed in the informal sector or are completely devoted to child-rearing and household activities. In addition, imbalances in social statuses such as inequality in production roles and opportunities, sexual abuse, a lack of money to meet basic requirements, and gender inequality both within and outside the house [7]. Furthermore, the children of young married girls face extreme hunger, undernutrition, and poor health.

According to a recent report from UNICEF, COVID-19 will result in an additional 10 million girls' marriages as a youngster by the year 2030. This is related to COVID-19 restriction measures, lockdowns, closing of schools, a higher rate of teenage pregnancy, interruption of programs that are working on child marriage, and instability in the economy. Furthermore, the COVID-19 pandemic and physical distancing procedures may have had an impact on vital registration systems, such as marriage and newborn registration, driving child marriages underground and disrupting data gathering on new child marriages [8].

### Child marriage in Ethiopia

Over the last decade, Ethiopia has experienced one of the world's fastest child marriage rate reductions. Efforts in girls' schooling, increased awareness of the legal age of marriage, and community activities emphasizing the economic and health impact of child marriage and teenage motherhood have resulted in a one-third reduction in rates [9], and in addition, early adolescent marriage is also declining at a fast rate.

According to the 2016 Ethiopian DHS (EDHS), only 5.7 percent of girls aged 15-19 were married before the age of 15, relative to 14.1 percent of women aged 20-24. By the age of 18, 40.3 percent of women in their 20s and early twenties had married, relative to 49.3 percent of women in their late twenties and early thirties [10]. Despite advances in the reduction, early marriage is still quite widespread among Ethiopian women. In 2016, the Central Statistical Agency (CSA) found that more than half of Ethiopian women (58%) married before they became 18 years old. About 11 percent of married women are in polygamous relationships, and the proportion of women who married young in polygamous families is higher than in monogamous homes [10]. Given Ethiopia's cultural and religious diversity, as well as its underdevelopment, evidence suggests that the risk of child marriage is not consistent and that more difference is likely. Ethiopia's diversity in culture and religion, as well as its underdevelopment, has resulted in a disparity in the prevalence of child marriage among the different regional states of the country. The recent EDHS revealed that the median age at first marriage in Ethiopia differs among regional states. Differences in ethnicity, language, urban-rural residency, and disparities in educational level were reported as the contributing factors [10]. The prevalence of early marriage among reproductive-age women varies significantly by region, ranging from 87 percent in the Amhara region and 26 percent in the Addis Ababa city administration [11,12]. Early marriage hotspots have been found in the Amhara, Tigray, and Afar regions.

Early marriage in Ethiopia includes promissory marriage, in which parents make a verbal agreement to have their children marry as early as infancy or even during childbirth, child marriage, in which children below the age of ten are married, and marriage during adolescence, in which children aged 10 to 15 are married, are all examples of early marriage in Ethiopia [11]. Girl bride is typically taken to her in-laws right after the marriage ceremony; but, in some circumstances, the parents agree that the girl should remain with her family till she is grown enough to start living with her spouse. Husbands are typically significantly higher in age than their younger brides [12,13].

Early marriage is a betrayal of a child's basic human rights. “Child marriage and betrothal of girls and boys shall be forbidden,” says Article 21 of the African Charter on the Rights and Welfare of the Child, and “Effective measures, including legislation, shall be adopted to establish the minimum age of marriage to be 18 years.” In addition, the Maputo Protocol on the Rights of Women in Africa (October 2005) and Ethiopia's recently approved criminal legislation (2005) recognize that women must be 18 years old to marry, and that marriage can only take place with both parties' full permission [3]. Unfortunately, many rural populations have poor awareness of and respect for the law. It is critical to comprehend the societal and family pressure that led parents to marry off their daughters [14].

### Determinants of child marriage in Ethiopia

There are a variety of factors that contribute to child marriage in Ethiopia. To mention the common ones, the educational status of women and their parents, household size of the family, location, and household economic position [15,16], first marriage decision, religious status [3], awareness to the optimal marital age, knowledge about accusations of teenage marriage [16], and exposure to news/media [17]. Marriage is also a deeply ingrained practice throughout most communities in Ethiopia. For example, in a rural part of the Amhara regional state, cultural and social understanding is that being a virgin until the girl marries is highly cherished; however, a girl who is unmarried over the age of 14 is frequently stigmatized. As a result, their children are forced to marry before the age of 14. Furthermore, young women in the Afar regional state are undervalued, have little or no influence over resources, and have limited power to make decisions even at the household level and outside, especially in their own lives. Young women are pushed to marry at a young age as a consequence of such serious cultural practices of marriage [13]. The following are the reasons for getting married (in order of significance) according to a study conducted by Pathfinder International/Ethiopia on “the incidence, causes, and personal and social implications of early marriage in the Amhara region”. 1. “It's a local custom.” 2. “It helps to strengthen bonds” 3. “For prestige” 4. “Difficult to get married if older” 5. “The family will be a victim of gossip” 6. “To earn dowry” 7. “To protect virginity and avoid premarital affairs” [18].

### Early marriage in Ethiopia: why does it persist?

The majority of nations have legislation prohibiting child marriage. The laws, however, are overshadowed by cultural, social, and economy-related challenges that play a significant role in the continuation of such inhuman and violent practices. In many developing countries, the frequently cited reasons for the persistence of early marriage are traditional customs, poor economic status, religious issues, and low educational level [19,20].

### Economic factors

In order to receive the dowry/cattle from the husband or from his family as a bride's price, many young girls and boys suffer such a discriminatory and harmful practice. Furthermore, to begin earning future return help from family, attach with “a financially better off or a better social standing“ family, and reduce the financial dependence of children, early marriage among the pastoral community of Ethiopia is highly supported [21]. Poor families are well-known for having insufficient financial resources to invest in their children's education, health care, or even food. As a result, they would resort to early marriage as a short-term and limited-quantity solution to their financial problems [3].

### Social reasons

Child marriage in Ethiopia is encouraged by seriously ingrained tradition, religious dogmas, socio-cultural stands, and values, especially in the Muslim societies of Ethiopia. Child marriage is acknowledged as a common occurrence throughout the country, regardless of the degree to which it occurs. It is sometimes promoted since it is thought to prevent the youngster from losing his or her virginity, abduction, and undesired pregnancy [22]. Religious and traditional customs hinder the legislation and regulations of child marriage practices at international, regional, and country levels. For example, Muslim adherents who promote early marriage justify that they are practicing it since their religion/the shariah permits the act. Practice child marriage argues that they do so because their religion, or the Shariah, permits it. They also stated that not practicing child marriage would mean disobeying/against their religion and religious dogmas. As a result, such communities in the population promote teenage marriage for religion-related reasons, despite the fact that such bigoted customary practices are prohibited by law [22].

### The impacts of early marriage in Ethiopia

If one examines the implications of early marriage critically, it´s at its most repulsive (disgusted form). Marriage at an early age is associated negatively with educational attainment, the health of the mother, combating the economic crisis, and other issues related to the well-being of women and girls [12]. As a result, the consequences of early marriage in Ethiopia are explored as follows.

### Psychological impact

If a youngster gets married during her teens, she will be vulnerable to emotional and psychological problems. When they are subjected to forced sexual intercourse, their adolescence is hammered, and their rights to personal growth are taken away [13]. The victims of early marriage are frequently not happy and commonly separated from the community they are living with. Premature sex and childbirth also result in psychological and mental shocks and other hazards. Most of these aches and pains either compelled the young woman to flee to her family's house or caused her to endure misery, which she communicated through excessive quiet and profound tears in front of her mother-in-law or alone. The saddest element of this tragedy is that women get assaulted on two sides - by their own families, their husbands or in-laws, or husbands fleeing their homes to avoid the upset [22]. Women who marry as youngsters may be more vulnerable to mental illness because they are exposed to a number of interconnected dangers, as the structural theories suggest. Their sensitivity to mental diseases may be exacerbated by the difficulties they confront as a result of their age and developmental stage, as well as their gender, marital status, socioeconomic background, and the interconnections among these factors. Early marriage necessitates a rapid move into gendered adult roles and responsibilities, which a young girl may not be developmentally equipped to handle [22].

### Denial of social services

Early marriage has a negative consequence, depriving them of opportunities for social services to immaturely married teens, in addition to the emotional and psychological effects. These girls' fates include dropping out of school, not having an opportunity for appropriate health services, not having access to adequate health care, being limited in mobility from one location to another, and being unable to have communication and networks. They are used as reproductive and domestic household labor instead of being integrated into social services and resources. These young married girls are solely responsible for raising their children, especially in rural Ethiopia [11].

Girls' right to education is profoundly harmed by child marriage. Early married children in Ethiopia, according to various scenarios, do not have the right to prepare for their future proper life pathways from childhood to maturity. The victims don't have the authority to make decisions in their own right. It is always a burden on her parents, the elderly, and society. According to certain studies, the behavior and perception of educating girls have shifted slightly. However, it is still thought that investing in a girl entails investing in someone else's property, which ultimately belongs to that person. Similarly, some parents worry about not educating their children since they believe they will soon leave the family and work for their husbands or their parents rather than themselves [13].

### Impact on reproductive health

Thousands of adolescent girls have passed away as a result of early pregnancy and delivery. Similarly, a report from UNICEF revealed that each year, over 70,000 young girls who are married between the ages of 15 and 19 years die as a result of pregnancy and childbirth problems [1]. Early pregnancy and fertility were also associated with poor outcomes such as obstetric fistula, excessive bleeding, HIV/AIDS contamination, and other sexually transmitted illnesses, according to the analysis [23]. Because early marriage occurs before the girl's body is mature enough for intercourse and to conceive a pregnancy or start childbearing, it is frequently followed by a very high rate of death and morbidity. As a result, those girls who were aged below 15 and died during childbirth were five times higher compared to married young women aged less than 20 years [24]. To sum up, it is not surprising that early marriage is associated with a high rate of antenatal and postpartum complications and death.

### Risk of treat/violence

Married teens who are under the age of 18 are not considered wives. They are excluded from family planning, conversations, and decision-making. Her spouse despises her and constantly hits, abuses, and humiliates her. She also has to deal with domestic violence regularly. In the meantime, due to her low economic and educational status, she is unable to leave the marriage. According to a UNICEF report, some men are also alcoholics who assault their spouses physically. Coercive sex, intimidation, and slapping are examples of this violence [25]. She may choose to leave that environment because of the hostility, but she will fall into severe poverty because she is responsible for her children and has a limited ability to create cash for both her children and herself. As a result, marriage at an early age is the leading source of feminization of poverty among young married women and their children [25]. Intimate partner violence is more common among women in these relationships than among their elder married peers [26]. Because of their lack of agency, young brides seem to be more prone to believe that their partners are right in assaulting them, making them more vulnerable to harassment and less ready to leave horrible situations [27]. Furthermore, these women's developmental period, loss of teenage years, and characteristics connected with child marriages, such as loss of personal independence and agency, forced sexual interactions, and higher exposure to intimate partner violence, may put them at risk for mental illnesses and decreased well-being [27].

### Migration

Migration is another big consequence of early marriage. When children can no longer bear the sufferings and traumas, they migrate to adjacent nearby places or outside nations, including the Mideast, with brokers´ assistance, without the agreement of their husbands, in-laws, or parents. Unfortunately, people may have a life that is worse than their homes in these regions. As domestic employees, they are treated inhumanely. Young girls are obliged to do intense work continuously for extremely long periods with no access to any interaction with their families and without networks like Saudi Arabia, Yemen, and Qatar, for example [22]. They suffer sexual assault including rape, sex trafficking, criminality, beatings, and death, and they are usually cheated by agents in their new destinations. These girls have also been known to die by suicide as a way of avoiding fear and anxiety. As this is happening, domestically and internationally, actors/stakeholders are failing to develop a solid and effective law to combat such criminal activity [28].

### Conclusion

In Ethiopia, the practice of marriage at earlier ages is influenced by strongly held convictions and customs which are often very difficult to change and obtain a decent amount of disapproval. The development of successful strategies to alleviate the concerns of parents can be enhanced by a thorough examination and understanding of the pressures they experience. The long-term goal of improving women's status in Ethiopia includes strengthening their personal rights to reproduction and increasing their ownership and access to resources to combat poverty and insecurity. Moreover, increasing their capacity to make decisions within family and community firmly establishes their value as equal participants in the growth and members of society. Although the country has shown an overall, reduction in the prevalence of child marriage, the problem is still striking and demands the attention of all stakeholders who are working on children’s and adolescents' health and well-being. The most challenging side of early marriage in Ethiopia is its link with diverse and highly deep-rooted issues such as religion, culture, ethnicity, and other conditions. Examining the customs surrounding child marriage, and involving society, families, and young people in the planning, implementation, and evaluation of all programs concerning child marriage is critical. Social and behavior change communication, including educating the community about the consequences of early marriage, involving religious leaders, community leaders, civil servants, and association leaders in all aspects of programs on child marriage, and establishing links/cooperation among schools, teachers, and students are paramount. To make the eradication of early marriage a reality, strengthening the role of the legal system, such as providing training to the policemen, and the prosecutors, and creating robust support mechanisms to encourage girls to stay in school. Furthermore, it is critical to pay attention to the variation in different ethnolinguistics, religions, and residences and avoid “a one size fits all” strategy. Context-specific approaches will also help to realize the benefit of interventions on child marriage on the attainment of the national commitment to eliminate child marriage and the global commitment, Sustainable Development Goal 5.3, to eliminate child marriage by the year 2030.

## References

[1] UNICEF (2014). Division of Communication. 25 years of the Convention on the Rights of the Child?: is the world a better place for children?.

[2] UNICEF (2018). Child marriage is a violation of human rights. but is all too common.

[3] Mitiku Y, Kiffle D, Siyoum D, Birlie B (2018). Determinants of time to first marriage among rural women in Ethiopia. Biomed Stat Inform.

[4] Erulkar A (2013). Early marriage, marital relations and intimate partner violence in Ethiopia. International perspectives on sexual and reproductive health.

[5] de Groot R, Kuunyem MY, Palermo T, Osei-Akoto I, Adamba C, Darko JK (2018). Child marriage and associated outcomes in northern Ghana: a cross-sectional study. BMC Public Health.

[6] Herliana BR, Utami NW, Kurniati DP (2018). Early marriage practices and the health impacts on female adolescent health in Central Lombok: a qualitative study. Public Health and Preventive Medicine Archive.

[7] Mutgan S (2014). Trends in Early Marriage in Shashemene. Ethiopia.

[8] UNICEF (2021). COVID-19. A treat to progress against child marriage.

[9] Harper C, Jones N, Marcus R, Bantebya G, Ghimire A (2018). Empowering adolescent girls in developing countries.

[10] Central statistical agency [Ethiopia] and ICF International: Ethiopia demographic and health survey (2016). Addis Ababa (Ethiopia): Central Statistical Agency [Ethiopia] and ICF International.

[11] Asrese K, Abebe M (2014). Early marriage in south Wollo and east Gojjam zones of the Amhara region, Ethiopia. Humanities and Social Sciences.

[12] Bezie M, Addisu D (2019). Determinants of early marriage among married women in Injibara town, North West Ethiopia: Community-based cross-sectional study. BMC Womens Health.

[13] Alem AZ, Yeshaw Y, Kebede SA, Liyew AM, Tesema GA, Agegnehu CD (2020). Spatial Distribution and Determinants of Early Marriage among Married Women in Ethiopia: A spatial and Multilevel Analysis. BMC Womens Health.

[14] John NA, Edmeades J, Murithi L, Barre I (2019). Child marriage and relationship quality in Ethiopia. Cult Health Sex.

[15] Workineh S, Kibretb GD, Degu G (2015). Determinants of early marriage among female children in Sinan district, Northwest Ethiopia. Health Science Journal.

[16] Hotchkiss DR, Godha D, Gage AJ, Cappa C (2016). Risk factors associated with the practice of child marriage among Roma girls in Serbia Health and human rights of marginalized populations. BMC Int Health Hum Rights.

[17] Rumble L, Peterman A, Irdiana N, Triyana M, Minnick E (2018). An empirical exploration of female child marriage determinants in Indonesia. BMC Public Health.

[18] Alemu B (2008). Early marriage in Ethiopia: causes and health consequences. Exchange on HIV and AIDS. Sexuality and Gender.

[19] Hervish A, Feldman-Jacobs C (2011). Who speaks for me? Ending child marriage. Population Reference Bureau.

[20] Ngusu M, Wondafrash B, Segni H, Gurmessa A (2015). Knowledge, attitude and practice of family planning methods among laboring mothers in Adama hospital, Oromia region, Ethiopia. J Womens Health Issues Care 4.

[21] Paulos M (2006). Early marriage in Ethiopia. Ethiopian Women Lawyers Association. Addis Ababa, Ethiopia.

[22] Khaleel YK, Alkhaldi AN (2017). Enterprise resource planning (ERP) model for small and medium sized manufacturing firms based on UML. International Journal of Information, Business and Management.

[23] Fernandes-Alcantara AL (2014). Missing and exploited children: Background, policies, and issues.

[24] USAID (2012). Ending Child Marriage & Meeting the Needs of Married Child.

[25] UNICEF (2001). Early Marriage: Child Spouses, Florence.

[26] Kidman R (2017). Child marriage and intimate partner violence: a comparative study of 34 countries. International journal of epidemiology.

[27] Nasrullah M, Oraka E, Chavez PR, Johnson CH, DiNenno E (2017). Factors associated with condom use among sexually active US adults, National Survey of Family Growth, 2006-2010 and 2011-2013. The journal of sexual medicine.

[28] Jones N, Tefera B, Stephenson J, Gupta T, Pereznieto P, Emire G (2014). Early marriage and education: the complex role of social norms in shaping Ethiopian adolescent girls´ lives. Country Report: Shaping policy for development.

